# A pilot study of invasive vagus nerve stimulation in Chinese patients with Alzheimer’s disease: preliminary results on safety, cognition, and biomarkers

**DOI:** 10.3389/fnins.2026.1934496

**Published:** 2026-09-29

**Authors:** Yingchuan Chen, Tenghong Lian, Dongmei Luo, Rujin Wang, Aolan Li, Dongmei Gao, Tingting Du, Qin Xu, Anxin Wang, Fangang Meng, Anchao Yang, Wei Zhang, Jianguo Zhang

**Affiliations:** 1 Department of Neurosurgery, Beijing Tiantan Hospital, Capital Medical University, Beijing, China; 2 Center for Cognitive Neurology, Department of Neurology, Beijing Tiantan Hospital, Capital Medical University, Beijing, China; 3 Department of Functional Neurosurgery, Beijing Neurosurgical Institute, Capital Medical University, Beijing, China; 4 Department of Epidemiology, Beijing Neurosurgical Institute, Capital Medical University, Beijing, China; 5 China National Clinical Research Center for Neurological Diseases, Beijing Tiantan Hospital, Capital Medical University, Beijing, China; 6 Department of Clinical Epidemiology and Clinical Trial, Capital Medical University, Beijing, China

**Keywords:** Alzheimer’s disease, cognitive function, inflammation, psychological symptom, vagus nerve stimulation

## Abstract

**Introduction:**

Alzheimer’s disease (AD) is a progressive neurodegenerative disorder with no curative therapies, and vagus nerve stimulation (VNS) has emerged as a promising neuromodulatory approach. This prospective, open-label pilot study aimed to evaluate the feasibility, safety, and preliminary cognitive, psychological, inflammatory, and AD biomarker effects of invasive VNS (iVNS) in Chinese AD patients.

**Methods:**

Five AD patients (mean age: 62.4 ± 4.0 years) underwent iVNS implantation, with assessments at baseline, 3, and 6 months postoperatively (one patient did not complete the 6-month follow-up). Cognitive function (MMSE, CDR, VFT, BNT), psychological/behavioral symptoms (CMAI, HAMA, HAMD), sleep/daily-living status (ESS, ADL), serum inflammatory factors, and AD biomarkers were measured.

**Results:**

Among cognitive outcomes, MMSE increased by 1 point at both follow-ups and VFT rose by 5.2 and 4.25 points at 3 and 6 months, respectively, although none of these changes reached statistical significance; global CDR remained unchanged. HAMA and HAMD scores decreased, and ESS scores declined by 25–27%, without reaching significance. Serum pro-inflammatory Eotaxin and CCL5 were significantly reduced (*p* = 0.026 and *p* = 0.045, respectively), whereas CXCL10 levels showed a non-significant numerical increase. Although the Aβ42 and Aβ42/Aβ40 ratio showed numerical increases (by 126.4% and 75.3%) and pTau181, pTau217, and NfL showed numerical decreases, these changes were not statistically significant. Only transient hoarseness was reported.

**Discussion:**

These preliminary findings suggest that iVNS is feasible and associated with early signals of cognitive, psychological, inflammatory, and biomarker changes in AD patients, which warrant confirmation in larger, randomized, sham-controlled trials.

## 1 Introduction

Alzheimer’s disease (AD) stands as one of the most pressing global health challenges of the 21st century, characterized by progressive cognitive decline and behavioral disturbances (Jia et al., 2020). Pathologically, the disease is defined by the accumulation of amyloid-beta (Aβ) plaques and tau-associated neurofibrillary tangles, alongside widespread neuronal loss and synaptic dysfunction (Andrade-Guerrero et al., 2023). Despite decades of intensive research, current pharmacological interventions—including cholinesterase inhibitors and NMDA receptor antagonists—only provide modest symptomatic relief and fail to halt disease progression (Rajanna et al., 2025).

The emergence of neuromodulation therapies has opened new therapeutic avenues for neurodegenerative disorders, with vagus nerve stimulation (VNS) emerging as a particularly promising approach for AD treatment (Vargas-Caballero et al., 2022). VNS has already demonstrated clinical efficacy in drug-resistant epilepsy and treatment-refractory depression, with over 125,000 patients worldwide having received implantable VNS therapy for epilepsy (Afra et al., 2021). The rationale for applying VNS to AD stems from the vagus nerve’s extensive anatomical connections to brain regions critical for memory and cognition, such as the locus coeruleus (LC), nucleus basalis of Meynert (NBM), hippocampus, and neocortex (Saggu et al., 2025; Vargas-Caballero et al., 2022).

Initial clinical evidence for VNS in AD came from small, open-label pilot studies that provided proof-of-concept for this therapeutic strategy. These pioneering investigations yielded promising results, with 70% of patients showing improvement or stability in cognitive measures after 3 months of treatment (Sjögren et al., 2002). Additionally, researchers have explored the efficacy of transcutaneous auricular VNS (taVNS) in patients with mild cognitive impairment (MCI), revealing a significant difference in overall MOCA-B scores between the taVNS and sham groups (Wang et al., 2022).

A key advantage of invasive VNS (iVNS) over taVNS lies in its direct, targeted, and programmable stimulation of core vagal pathways, which may enable AD patients to derive greater benefits and more stable therapeutic effects. However, the efficacy of VNS in treating Chinese AD patients remains unclear, and the precise mechanisms underlying VNS’s therapeutic effects require further investigation. To address these gaps, we conducted a prospective, open-label pilot study on Chinese AD patients to explore these questions.

## 2 Materials and methods

### 2.1 Study participants and ethical considerations

This study strictly adhered to the Declaration of Helsinki. Its protocol—encompassing recruitment, intervention, and data collection—was formally approved by the Ethics Committee of Beijing Tiantan Hospital (Approval Number: KY2023-238-03; Clinical trial registration number: ChiCTR2400086775). Participants were recruited via a standardized protocol and enrolled after meeting eligibility criteria and providing written informed consent (Details in Supplementary material). All participants were fully informed of study details and their right to withdraw freely. Data confidentiality was strictly maintained, and the ethics committee supervised all procedures to protect participants’ rights and safety. The inclusion and exclusion criteria are provided in the Supplementary material.

### 2.2 Surgical procedure and programming

The device implantation procedure was consistent with a previously described protocol (Liu et al., 2022). Detail was shown in Supplementary material.

Patients returned to the hospital 2 weeks after VNS system implantation for initial programming, which was conducted in standard mode (magnet mode excluded). Initial parameters were set as follows: frequency 30 Hz, pulse width 250 μs, output current 0.5 mA, stimulation cycle 30 s, and stimulation interval 5 min. During activation, patients were closely monitored for adverse reactions such as dizziness, palpitations, nausea, headache, paresthesia, hoarseness, cough, chest tightness, dyspnea, and bradycardia; if reactions occurred, the current was reduced to the maximum tolerated level. Over the subsequent 14 days, patients attended 2–3 follow-up visits, with the current gradually increased by 0.1–0.2 mA per visit (other parameters unchanged) and reactions re-evaluated. Final parameters were titrated to 30 Hz, 250 μs, 1.2 mA (or maximum tolerance), with 30 s of stimulation and a 5-min interval, ensuring no severe discomfort. For patients sensitive to side effects, follow-up frequency was increased and the amplitude of current adjustment was reduced to balance safety and efficacy.

### 2.3 Clinical evaluation

Experienced neurologists assessed patients’ clinical status at baseline and postoperatively using standardized neurological assessment scales. A battery of clinical and functional assessments was conducted at baseline (within 1 month prior to VNS system implantation), 3 and 6 months post-implantation. These assessments included the Mini-Mental State Examination (MMSE), Boston Naming Test (BNT), Verbal Fluency Test (VFT), Clinical Dementia Rating Scale (CDR), Cohen-Mansfield Agitation Inventory (CMAI), Hamilton Anxiety Rating Scale (HAMA), Hamilton Depression Rating Scale (HAMD), Epworth Sleepiness Scale (ESS), and Activity of Daily Living Scale (ADL). To minimize medication-related confounding in within-patient comparisons, each participant’s medication regimen was required to remain stable from 1 month before enrolment through the 6-month postoperative follow-up.

### 2.4 Disease biomarker and inflammatory factor measurement

Serum samples were collected at two time points: preoperatively and 6 months postoperatively. At each time point, 4–5 mL of peripheral venous blood was drawn into sterile serum coagulation tubes and centrifuged at 4,000 rpm for 10 min within 2 h of collection. The isolated serum was then aliquoted and stored at −80 °C until subsequent analysis. Serum concentrations of cytokines and chemokines were measured using the Cytokine & Chemokine 34-Plex Human ProcartaPlex kit (Invitrogen, Waltham, MA, USA) in conjunction with a Bio-Plex 200 microplate reader (Bio-Rad Laboratories, Hercules, CA, USA), strictly following the manufacturers’ instructions. The level of serum Aβ42, Aβ40, pTau217, pTau181 and neurofilament light chain (NfL) concentrations were quantified on the fully automated Lumipulse G1200 platform via available kits (Fujirebio Europe, Ghent, Belgium).

### 2.5 Data analysis

Continuous variables are presented as mean ± standard deviation (SD). All statistical analyses and graphical visualizations were performed using GraphPad Prism 10.1 (GraphPad Software, Inc., San Diego, CA, USA). For each endpoint, the normality of the paired differences was first assessed using the Shapiro–Wilk test. Endpoints whose paired differences met the normality assumption — all inflammatory cytokines and AD biomarkers, as well as MMSE, BNT, ADL, ESS, HAMA, HAMD and CMAI — were compared using paired-samples *t*-tests. Endpoints whose paired differences violated normality – specifically the Clinical Dementia Rating (CDR) global score and the Verbal Fluency Test (VFT) at both 3 and 6 months – were analysed with the non-parametric Wilcoxon signed-rank test. If data were lost during follow-up, complete-case analysis was conducted, excluding pairs with missing values. Because multiple endpoints were tested, we report uncorrected (nominal) p values and discuss the implications of multiple-comparison correction. A *p*-value < 0.05 was considered nominally statistically significant.

## 3 Results

### 3.1 Patient characteristics and programming parameters

Five AD patients were recruited and provided written informed consent. All patients were diagnosed with AD using positron emission tomography (PET, assessing both Aβ and tau) or cerebrospinal fluid (CSF, assessing both Aβ and tau). The cohort included both male and female patients, with a mean age of 62.4 ± 4.0 years and mean disease duration of 5.8 ± 1.9 years. VNS stimulation was programmed according to the protocol described above, with a mean final stimulation current of 1.16 ± 0.09 mA (Table 1). Only mild adverse events were observed; some patients experienced transient hoarseness during initial programming, with no other adverse events reported. All patients completed the 3-month postoperative follow-up; however, one patient did not attend the 6-month postoperative follow-up due to family reasons (Figure 1).

**TABLE 1:** Summary of patient demographics and current treatment.

Subject	Sex	Age (years)	Disease duration (years)	CDR	Medication (daily dose)	Stimulation parameter
Patient 1	Male	58	6	2	Donepezil 10 mg; GV-971 900 mg	30 Hz, 250 μs, 1.2 mA
Patient 2	Male	66	7	1	None	30 Hz, 250 μs, 1.0 mA
Patient 3	Female	62	3	2	Donepezil 10 mg	30 Hz, 250 μs, 1.2 mA
Patient 4	Male	59	5	2	Memantine Hydrochloride 20 mg, Olanzapine 5 mg, Pentoxifylline 800 mg	30 Hz, 250 μs, 0.5 mA
Patient 5	Female	67	8	0.5	GV-971 900 mg	30 Hz, 250 μs, 1.0 mA

**FIGURE 1:**
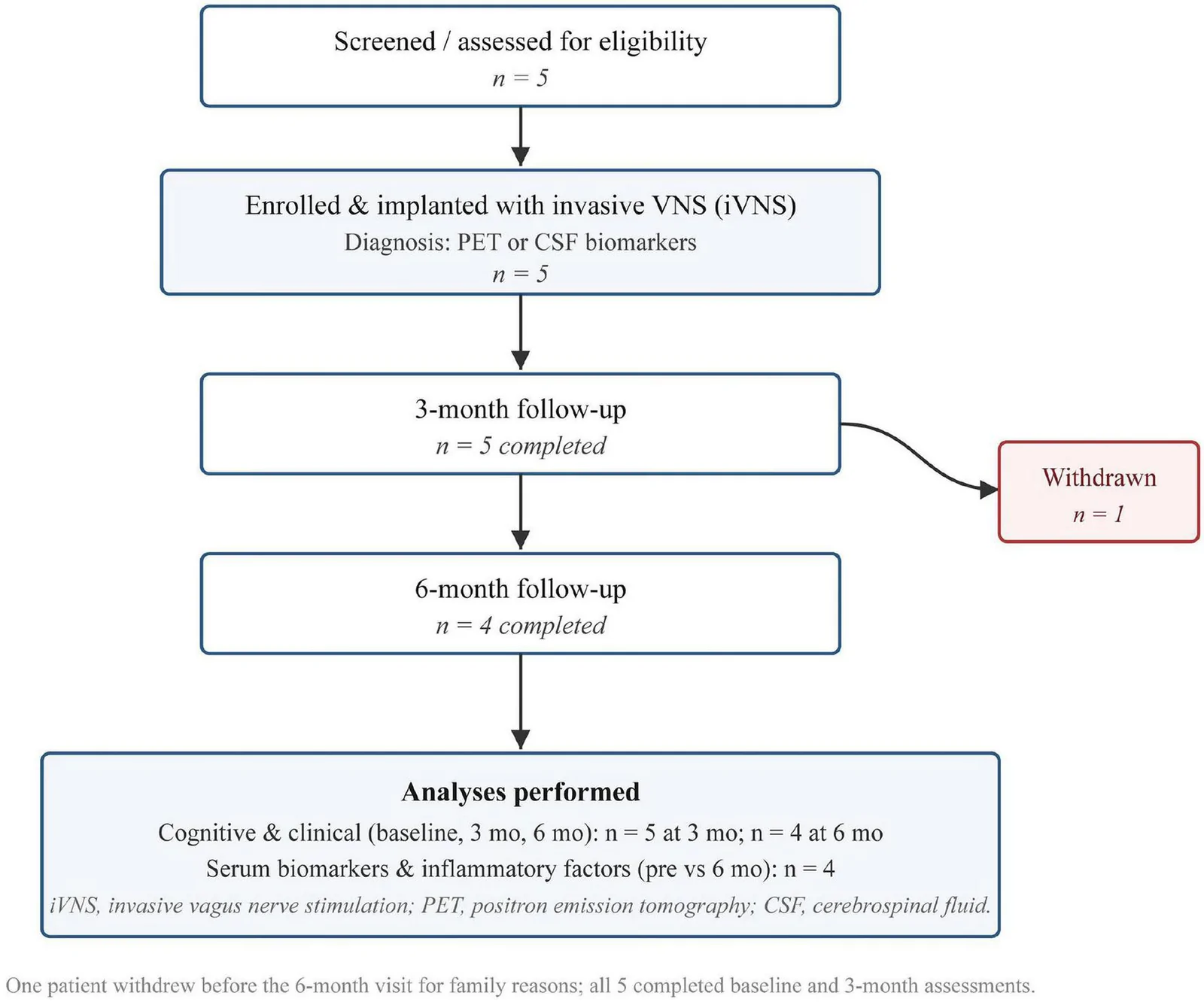
Participant flow diagram. All five screened AD patients (diagnosed by PET or CSF biomarkers) were enrolled and implanted with iVNS. All five completed the 3-month follow-up; one patient withdrew before the 6-month visit for family reasons, leaving four patients for the 6-month analyses. Cognitive and clinical outcomes were analysed at 3 months (*n* = 5) and 6 months (*n* = 4); serum biomarkers and inflammatory factors were compared between baseline and 6 months (*n* = 4).

### 3.2 Cognitive function outcomes

MMSE, VFT, and BNT were used to evaluate changes in cognitive function. MMSE scores increased by 1 point at both 3 months and 6 months postoperatively; these changes did not reach statistical significance (paired *t*-test, *p* = 0.561, mean difference = 1.00 [95% CI −3.39 to 5.39] at 3 months and *p* = 0.613, mean difference = 1.00 [95% CI −4.66 to 6.66] at 6 months; Cohen’s d_z ≈ 0.28 for both) (Figure 2A). Global CDR scores remained unchanged during the follow-up period (Wilcoxon signed-rank test, *p* = 1.000) (Figure 2B). The Wilcoxon signed-rank test indicated no statistically significant change in VFT (3 months: *p* = 1.000, Hodges-Lehmann median difference = −2.0 [95% CI −2.0 to 32.0]; 6 months: *p* = 1.000, Hodges’Lehmann median difference = −2.0 [95% CI −2.0 to 23.0]) (Figure 2C). BNT results showed a non-significant increase of 0.6 at 3 months postoperatively (paired *t*-test, *p* = 0.675, mean difference = 0.60 [95% CI −3.08 to 4.28], Cohen’s d_z = 0.20) and a non-significant decrease of 1 at 6 months postoperatively (*p* = 0.594, mean difference = −1.00 [95% CI −6.36 to 4.36], Cohen’s d_z = −0.30) (Figure 2D).

**FIGURE 2:**
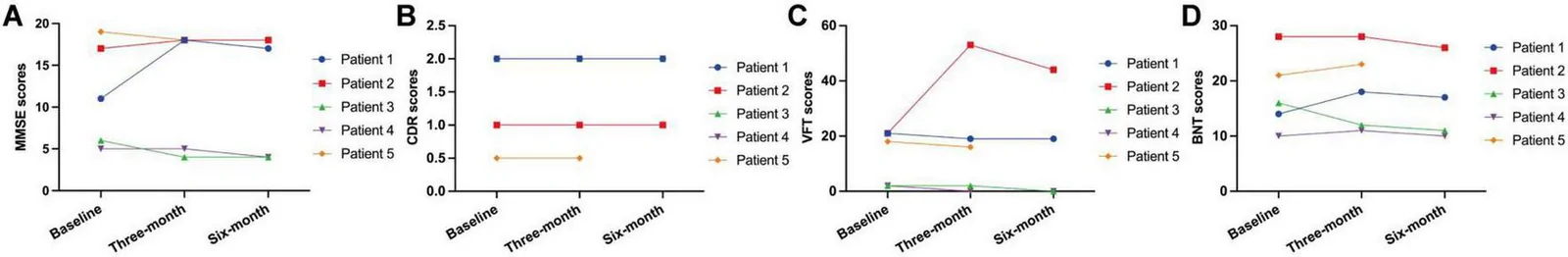
Cognitive function changes in AD patients after VNS. **(A)** MMSE scores increased by 1 point at both 3 and 6 months post-VNS (not significant). **(B)** Global CDR scores remained unchanged (Wilcoxon, *p* = 1.000). **(C)** VFT scores rose numerically by 5.2 at 3 months and 4.25 at 6 months post-operation, but the change was not statistically significant (Wilcoxon; normality violated). **(D)** BNT scores increased non-significantly by 0.6 at 3 months but decreased by 1 at 6 months after VNS intervention.

To contextualize these findings, we referenced public datasets: Miyakawa-Liu et al. reported a 1.2-point decrease in MMSE scores every 6 months (2.4 points per year) in AD patients (Miyakawa-Liu et al., 2022), while a meta-analysis estimated a 1.65-point decline every 6 months (3.3 points per year) (Han et al., 2000). Against this background, the observed ∼1-point MMSE stability in our cohort is within test–retest variability (Tombaugh and McIntyre, 1992) and below published minimal clinically important differences (MCID) for the MMSE—generally 1–3 points for clinically meaningful change in AD, increasing with disease severity, with stage-specific estimates of approximately 1.4–3 points in moderate-to-severe AD and ∼2 points in mild AD (Andrews et al., 2019; Muir et al., 2024)—and should therefore not be interpreted as definitive cognitive improvement.

### 3.3 Psychological and behavioral function outcomes

CMAI, HAMA, and HAMD were used to assess psychological state. CMAI scores decreased numerically by 2.8 at 3 months postoperatively (paired *t*-test, *p* = 0.473, mean difference = −2.80 [95% CI −12.63 to 7.03], Cohen’s d_z = −0.35) but increased by 0.75 at 6 months postoperatively (*p* = 0.909, mean difference = 0.75 [95% CI −18.41 to 19.91], Cohen’s d_z = 0.06) (Figure 3A). For HAMA, scores fell numerically by approximately 3.2 (*p* = 0.125, mean difference = −3.20 [95% CI −7.80 to 1.40], Cohen’s d_z = −0.87) and 1.25 (*p* = 0.667, mean difference = −1.25 [95% CI −9.61 to 7.11], Cohen’s d_z = −0.24) at 3 and 6 months postoperatively, respectively (Figure 3B). A similar non-significant trend was observed for HAMD scores, which decreased (*p* = 0.353, mean difference = −1.80 [95% CI −6.56 to 2.96], Cohen’s d_z = −0.47) at 3 months postoperatively and (*p* = 0.659, mean difference = −1.25 [95% CI −9.40 to 6.90], Cohen’s d_z = −0.24) at 6 months postoperatively (Figure 3C). For comparison, Guetin et al. reported a 0.26-point increase in HAMA scores over 6 months in AD patients (Guétin et al., 2009).

**FIGURE 3:**
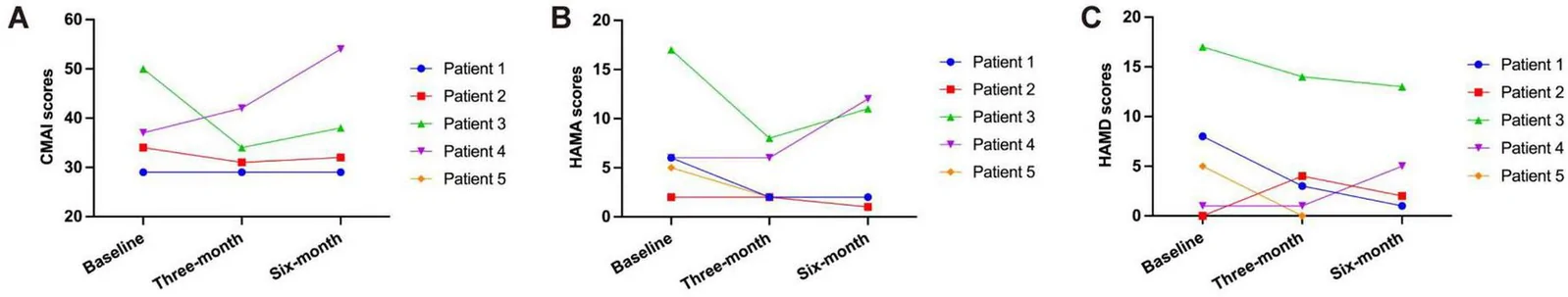
Psychological and behavioral function changes in AD patients post-VNS. **(A)** CMAI scores decreased non-significantly by 2.8 at 3 months but increased by 0.75 at 6 months. **(B)** HAMA scores dropped non-significantly by ∼3.2 and 1.25 at 3 and 6 months, respectively. **(C)** HAMD scores reduced non-significantly by 1.8 at 3 months and 1.25 at 6 months post-operation.

### 3.4 Quality of daily living and sleep outcomes

The ESS quantifies subjective daytime sleepiness to identify sleep disorders and monitor treatment effects. Specifically, ESS scores decreased numerically by 25% (paired *t*-test, *p* = 0.145, mean difference = −1.20 [95% CI −3.04 to 0.64], Cohen’s d_z = −0.81) at 3 months postoperatively and by 27.3% (*p* = 0.664, mean difference = −1.50 [95% CI −11.44 to 8.44], Cohen’s d_z = −0.24) at 6 months postoperatively (Figure 4A). For ADL scores, opposing non-significant trends were observed: scores decreased (*p* = 0.229, mean difference = −2.40 [95% CI −7.10 to 2.30], Cohen’s d_z = −0.64) at 3 months postoperatively compared to baseline, but increased (*p* = 0.547, mean difference = 1.50 [95% CI −5.56 to 8.56], Cohen’s d_z = 0.34) at 6 months postoperatively (Figure 4B).

**FIGURE 4:**
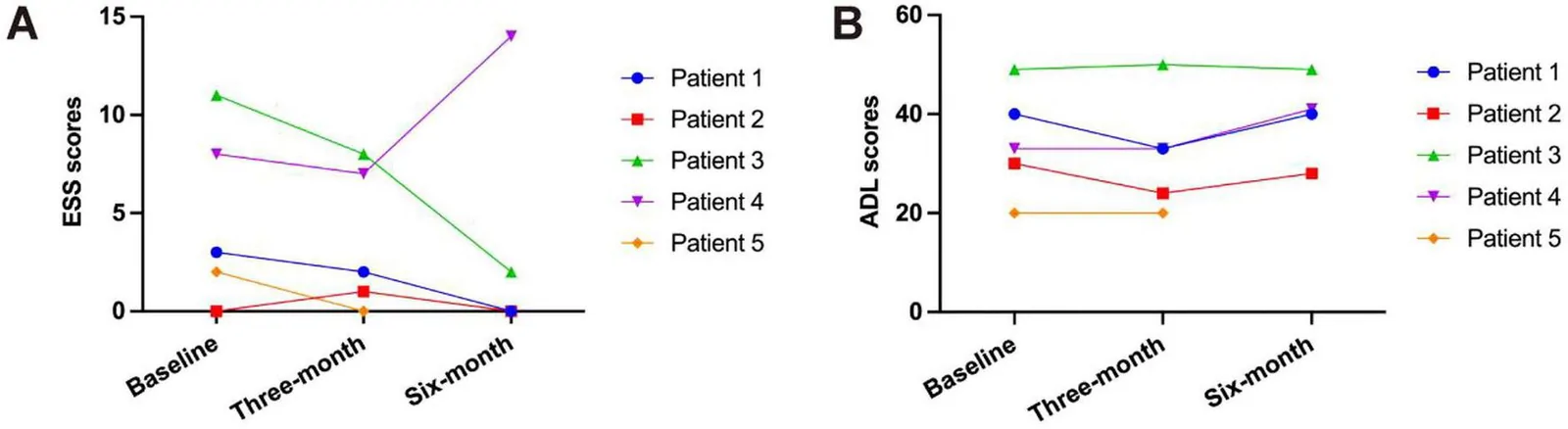
Changes in daily living quality and sleep status of AD patients after VNS. **(A)** ESS scores decreased non-significantly by 1.2 (25%) at 3 months and 1.5 (27.3%) at 6 months postoperatively. **(B)** ADL scores declined non-significantly by 2.4 points at 3 months but increased by 1.5 points at 6 months post-VNS.

### 3.5 AD biomarkers and inflammatory factors

Serum samples were collected from AD patients preoperatively and 6 months postoperatively. In AD, circulating Aβ, tau, and their phosphorylated forms play key roles in disease onset and progression; their abnormal levels in blood reflect cerebral pathological changes, correlate with cognitive decline, and serve as potential biomarkers for AD initiation and disease progression (Goedert, 2015).

Eotaxin and CCL5 levels were significantly decreased after VNS (Eotaxin: mean difference = −6.53 pg/mL [95% CI −11.58 to −1.47], *p* = 0.026, Cohen’s d_z = −2.05; CCL5: mean difference = −17.65 pg/mL [95% CI −34.49 to −0.80], *p* = 0.045, Cohen’s d_z = −1.67; paired *t*-test) (Figures 5A,B). In contrast, CXCL10 levels showed a numerical increase that did not reach statistical significance (mean difference = +7.65 pg/mL [95% CI −14.48 to 29.79], *p* = 0.351; Figure 5C). As for SDF-1α, a slight increment was detected, with no statistically significant difference (*p* = 0.890, mean difference = 15.05 pg/mL [95% CI −303.02 to 333.12], Cohen’s d_z = 0.08) (Figure 5D).

**FIGURE 5:**
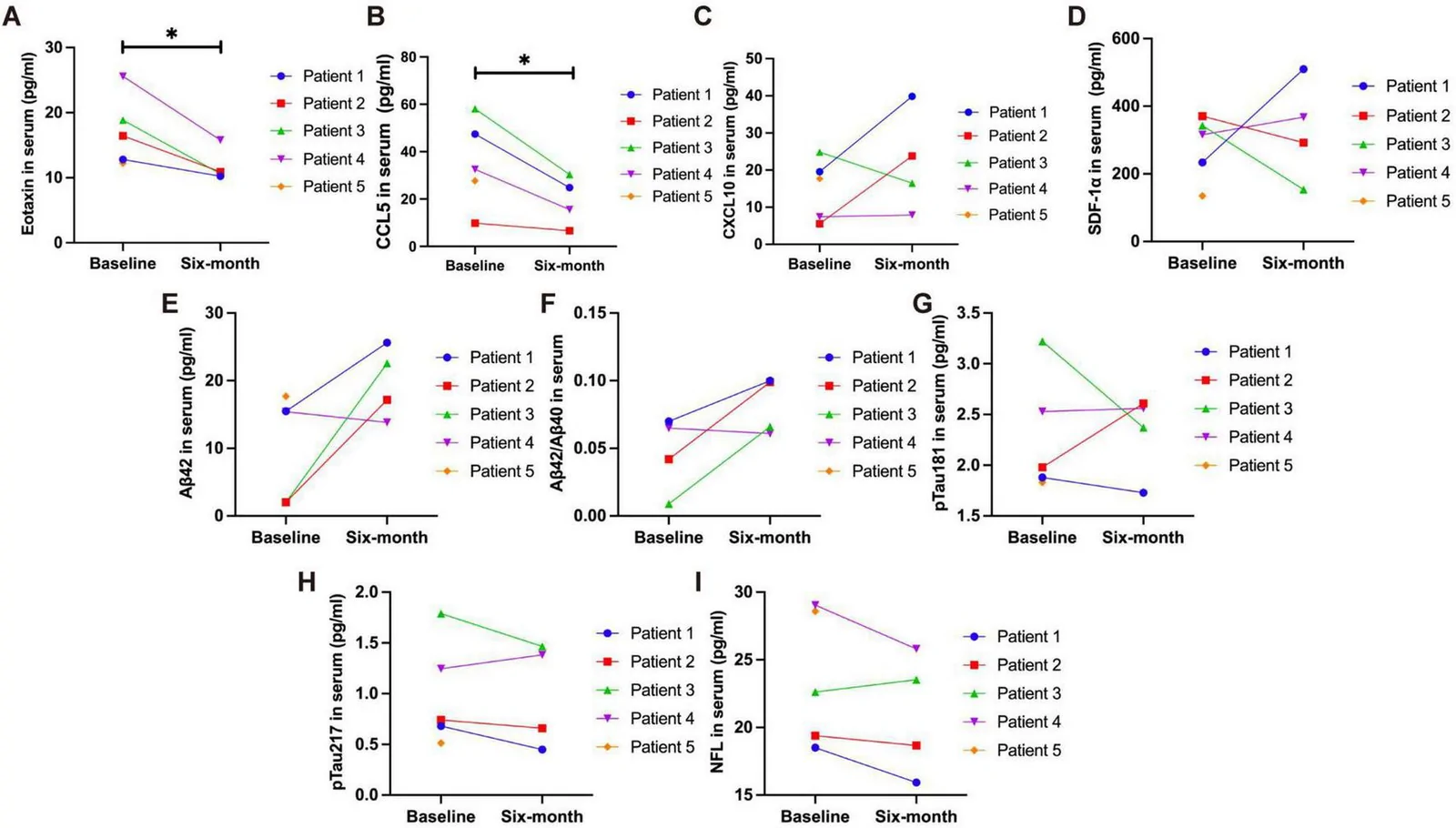
Changes in inflammatory factors and AD-related biomarkers after VNS. **(A,B)** Serum Eotaxin and CCL5 levels significantly decreased (Eotaxin *p* = 0.026; CCL5 *p* = 0.045). **(C)** CXCL10 level showed a non-significant numerical increase (*p* = 0.351), while SDF-1α showed a non-significant slight increment **(D)**. **(E–I)** Aβ42 and the Aβ42/Aβ40 ratio increased numerically by 126.4% and 75.3%, respectively (trend, *p* = 0.101 and *p* = 0.094); pTau181, pTau217, and NfL decreased numerically without significance. *p < 0.05.

Furthermore, we evaluated AD-related biomarkers and factors, including Aβ40, Aβ42, pTau181, pTau217, GFAP, and NfL. Although none reached statistical significance, we observed numerical changes: Aβ42 and the Aβ42/Aβ40 ratio increased numerically by 126.4% and 75.3% after VNS, respectively (Aβ42: mean difference = +11.05 pg/mL, 95% CI −3.94 to 26.05, *p* = 0.101; Aβ42/Aβ40: +0.035, 95% CI −0.011 to 0.081, *p* = 0.094; paired *t*-test; both trend-level) (Figures 5E,F). pTau181 and pTau217 decreased numerically by 3.5% and 11.2% (*p* = 0.798, mean difference = −0.08 pg/mL [95% CI −1.06 to 0.89], Cohen’s d_z = −0.14 and *p* = 0.300, mean difference = −0.13 pg/mL [95% CI −0.44 to 0.19], Cohen’s d_z = −0.62, respectively), and NfL decreased by 6.3% (mean difference = −1.41 pg/mL [95% CI −4.40 to 1.58, *p* = 0.231, Cohen’s d_z = −0.75) (Figures 5G–I).

## 4 Discussion

AD is a progressive neurodegenerative disorder with an extremely high global incidence (Jia et al., 2020). Currently, no curative treatments exist (Rajanna et al., 2025; Reynolds, 2019); VNS appears to be a potential treatment for AD, but further investigation is needed. In this prospective, open-label pilot study, we enrolled five AD patients who underwent iVNS and observed preliminary, mostly non-significant signals of cognitive stability, reduced anxiety/depression scores, reduced systemic inflammation (Eotaxin, CCL5), and altered AD biomarkers.

Neuromodulation refers to the targeted regulation of neural circuits via electrical or chemical stimulation (Denison and Morrell, 2022). Approximately 80% of vagal nerve fibers are afferent, carrying sensory information from peripheral organs to the central nervous system (Berthoud and Neuhuber, 2000). This extensive neural network makes the vagus nerve an ideal target for neuromodulation.

Pioneering invasive VNS studies in AD have shown promising results, with 7 patients remaining responders on both the ADAS-cog and MMSE after 6 months of treatment (Sjögren et al., 2002). A double-blind randomized trial confirmed that transcutaneous auricular VNS is safe and effective for mild cognitive impairment (MCI), observing improvements in overall cognitive function (assessed by MOCA-B) and memory (immediate and delayed recall via AVLT-H N5 and N7) (Wang et al., 2022). Dolphin et al. found that transcutaneous VNS is safe and tolerable in older adults with amnestic MCI, with no adverse hemodynamic effects and significant improvements in spatial navigation compared to baseline and sham (Dolphin et al., 2024). Consistent with these prior findings, our pilot observations are compatible with a potential signal of cognitive and psychiatric stabilization, although our cognitive changes did not reach statistical significance and must be confirmed.

The therapeutic effects of VNS in AD are mediated by complex neural circuits. The vagus nerve projects to the brainstem nucleus tractus solitarius (NTS) and spinal trigeminal nuclei, then connects monosynaptically to the LC—the primary source of cerebral norepinephrine (NE) (Galgani and Giorgi, 2023). Beyond the noradrenergic system, VNS also activates the central cholinergic system through projections to the nucleus basalis of Meynert (NBM) (Taylor et al., 2022). Our previous study found that NBM deep brain stimulation (DBS) improves short-term cognitive performance in patients with advanced AD, modulates multi-network connectivity patterns, and identifies the hippocampus as a key node within these networks (Jiang et al., 2022). Therefore, we hypothesize that VNS may exert therapeutic effects via a similar mechanism. Modulation of the cholinergic system represents another critical mechanism of VNS action in AD (Gargus et al., 2025).

Both NE and the cholinergic system exert anti-inflammatory effects through distinct but complementary mechanisms (Tracey, 2002; Borovikova et al., 2000; Li et al., 2025; Liu et al., 2018). VNS has been shown to modulate microglial function through multiple pathways: in aged APP/PS1 transgenic mice, brief non-invasive VNS induced significant morphological changes in microglia, shifting from a neurodestructive to a neuroprotective phenotype (Kaczmarczyk et al., 2017). Eotaxin exerts multiple effects on AD pathogenesis; it is considered a potential risk factor for AD development, and its plasma levels are correlated with the age of AD onset (Ivanovska et al., 2020). Cloned and expanded CD8^+^ T cells in the brain highly express CCL5, which binds to CCR5 on microglia, impairs microglial phagocytosis of Aβ, increases Aβ plaque deposition, and exacerbates early AD pathology (Ma et al., 2023). In this study, we found that VNS can modulate Eotaxin and CCL5 levels in the blood of AD patients, which may indicate selective anti-inflammatory modulation.

A previous study found that VNS reduces hippocampal Aβ1-40 and Aβ1-42 levels—the primary components of amyloid plaques–in APP/PS1 transgenic mice (Yu et al., 2023). We observed numerical increases in serum Aβ42 and the Aβ42/Aβ40 ratio after VNS (by 126.4% and 75.3%, respectively), although these changes were not statistically significant. We caution that Yu et al. measured Aβ in brain tissue of APP/PS1 mice, whereas we measured Aβ in serum; the relationship between central and peripheral Aβ is complex and not simply reciprocal, because peripheral Aβ arises from multiple sources and is governed by blood–brain-barrier dynamics and peripheral clearance. A rise in serum Aβ could alternatively reflect peripheral mobilization or altered clearance rather than reduced central burden. These cross-species, cross-matrix comparisons should therefore be interpreted with caution, and central Aβ/tau changes cannot be inferred from our peripheral measurements.

Notably, several endpoints exhibited opposing trajectories between 3 and 6 months: BNT improved at 3 months but declined at 6 months, whereas CMAI and ADL improved at 3 months but worsened at 6 months. These divergent trends, together with the loss of one patient to 6-month follow-up, indicate that the durability of any early signal remains uncertain in this small uncontrolled cohort. This underscores the preliminary nature of the findings and the need to assess durability in longer, controlled studies.

This study has several important limitations that constrain interpretation. First, the sample size was very small (*n* = 5, with *n* = 4 completing the 6-month follow-up), and the follow-up duration was limited, severely restricting statistical power, increasing susceptibility to outliers, and preventing reliable inference; the study should be regarded as an exploratory pilot rather than an efficacy trial. Second, there was no sham or concurrent control group and no blinding; consequently, placebo effects, learning/practice effects from repeated testing, disease heterogeneity, and differences in concomitant treatment cannot be excluded, and all conclusions must be substantially tempered. Third, the study population was heterogeneous with respect to disease severity (CDR 0.5–2), concomitant medications (donepezil, memantine, GV-971, olanzapine, pentoxifylline), and diagnostic confirmation (PET versus CSF biomarkers); Although medication regimens were required to remain unchanged from 1 month before enrolment through the 6-month postoperative follow-up, this heterogeneity remains a potential confounder. Given the small sample size, we could not perform meaningful stratified analyses by disease stage or medication status; future larger cohorts should examine whether VNS responses differ across AD stages and medication backgrounds.

## 5 Conclusion

These preliminary, open-label findings suggest that invasive VNS is feasible and associated with early signals of cognitive, psychological, inflammatory, and biomarker changes in Chinese AD patients. Definitive conclusions require confirmation in larger, randomized, sham-controlled, double-blind trials with stratified, balanced concomitant medications and longitudinal durability assessments.

## Data Availability

The original contributions presented in this study are included in this article/Supplementary material, further inquiries can be directed to the corresponding authors.
