# Supplementary material for: A pilot study of invasive vagus nerve stimulation in Chinese patients with Alzheimer’s disease: preliminary results on safety, cognition, and biomarkers
Source: Front Neurosci. 2026 Sep 29;20:1934496. doi: 10.3389/fnins.2026.1934496 (PMC13658621; doi:10.3389/fnins.2026.1934496)
Supplement: Supplementary file 1 [file Table_1.docx]

**Supplementary Materials**

**2.1. Study Participants and Ethical Considerations**

**Inclusion criteria:**

（1） Age: 50−80 years
（2） Meets the diagnostic criteria for AD established by the National Institute on Aging and the Alzheimer’s Association NIA-AA)
（3） Mild to moderate cognitive impairment, with a clinical cognitive rating scale (CDR) score of 0.5−2
（4） Stable use of the drug for at least 1 month without plans to change the medication within 6 months after randomization

**Exclusion criteria:**

（1） Dementia caused by other reasons: vascular dementia, central nervous system infections (such as AIDS, syphilis, etc.), Creutzfeldt-Jakob disease, Huntington’s disease and Parkinson’s disease, dementia with Lewy bodies, traumatic brain injury dementia, other physical factors (such as drug poisoning, alcohol poisoning, carbon monoxide poisoning, etc.), significant physical illnesses (such as hepatic encephalopathy, pulmonary encephalopathy, etc.), intracranial space-occupying lesions (such as subdural hematoma or brain tumor), endocrine system disorders (such as thyroid disease or parathyroid disease), and dementia due to vitamin deficiency or any other cause
（2） Serious or unstable disease, including cardiovascular, liver, kidney, gastrointestinal, respiratory, endocrine, neurological (AD-derived cognitive impairment excluded), psychiatric, immune or blood disorders, and other diseases that the investigator believes may have affected the analysis of this study, or life expectancy < 24 months
（3） A cancer history of less than 5 years, except for non-metastatic basal cell carcinoma and/or squamous cell carcinoma, cervical carcinoma in situ, nonprogressive prostate cancer, or other cancers with low risks of recurrence or spread
（4） Participants currently diagnosed with any primary mental disorder other than AD-induced cognitive impairment must be excluded if the investigator deems that the presence of such mental disorder or symptoms may have affected the interpretation of the (VNS) effects, cognitive assessment, or the ability of the participant to complete the study. Participants with a history of schizophrenia or other chronic psychiatric conditions must also be excluded.
（5） Participants who, according to the researchers’ judgment, have suicidal tendencies and are therefore considered to be at significant risk of suicide
（6） Illiteracy or insufficient education to complete the scale assessment
（7） A history of alcohol or drug abuse (excluding a history of smoking) within 2 years prior to the screening visit
（8） A history of multiple or severe drug allergies, obvious atopic allergy constitution, or severe hypersensitivity after treatment (including but not limited to severe polymorphic erythema, linear IgA dermatosis, toxic epidermal necrolysis, and/or exfoliative dermatitis) with a significant clinical significance
（9） Significant abnormalities that may be clinically significant and harmful to the patient, that affect the study or suggested evidence of other causes of dementia during screening (as determined by the investigator) during physical examinations or neurological examinations, as well as vital signs, electrocardiogram (ECG) or clinical laboratory tests
（10） Screening magnetic resonance imaging (MRI) results showing significant abnormalities suggesting another potential cause of progressive cognitive impairment, or findings with clinical significance that may have affected the participant’s ability to safely participate in the study. For example, more than two infarcts larger than 2 cm in diameter, as well as infarcts in critical areas such as the thalamus, hippocampus, internal olfactory cortex, parahippocampal cortex, angular gyrus, and other gray matter nuclei in the subcortical regions; a score of > 2 on the Fazekas Scale scale
（11） Any MRI contraindications, including claustrophobia, or the presence of an inoperable metal (ferromagnetic) implantation/cardiac pacemaker
（12） There are contraindications for VNS surgery, such as left vagus nerve injury, severe infection at the surgical site, severe heart, lung, liver, kidney, or other system dysfunctions that cannot tolerate general anesthesia surgery
（13） Currently participating in other interventional clinical trials, or any other type of medical study that is considered scientifically or medically incompatible with this study
（14） Other reasons that hinder the completion of this study, such as lack of stable caregivers
（15） Female subjects who are pregnant or planning to become pregnant

Staff members of the research center directly related to this study and/or their immediate family members. Immediate family members refer to spouses, parents, children, or siblings, whether biological or legally adopted relatives.

**2.2. Surgical Procedure and Programming**

Under general endotracheal anesthesia and antibiotic prophylaxis, patients were placed in a supine position with their head slightly turned to the right and extended to facilitate access to the left carotid sheath. The left vagus nerve was selected for implantation to avoid cardiac side effects associated with right vagus stimulation. A 4–5 cm skin incision was made along a natural crease for cosmetic purposes, and subcutaneous dissection identified the platysma, which was transected using electrocautery to expose the investing layer of the deep cervical fascia. Sharp dissection of this layer allowed blunt separation to the carotid sheath, where a blunt self-retaining retractor laterally retracted the internal jugular vein to expose at least 3 cm of the left vagus nerve (between the clavicle and mastoid, below the cardiac branches).

Next, a 4–5 cm left pectoral incision was created to form a subcutaneous pocket for the implantable pulse generator (IPG), with monopolar electrocautery (used only pre-implantation) to prevent hematoma. The lead connector was tunneled to the chest, and helical electrodes were wrapped around the vagus nerve below the cardiac branches (cathode cranial, anode middle, anchor caudal). Leads were secured to the fascia, connected to the IPG using a torque wrench, and functionally tested. After confirming proper functionality, the IPG was sutured to the pectoralis fascia, incisions were irrigated, and layered closure was performed with final safety checks.
